# The Mechanism by 18 RCTs Psychosocial Interventions Affect the Personality, Emotions, and Behaviours of Paediatric and Young Adult Cancer Patients: A Systematic Review

**DOI:** 10.3390/healthcare13101094

**Published:** 2025-05-08

**Authors:** Xiao Liu, Honglin Chen, Natalie Joubert, Heli Tiirola

**Affiliations:** Department of Social Sciences, Faculty of Social Science and Business Studies, University of Eastern Finland, FI-70210 Kuopio, Finlandnatalie.joubert@uef.fi (N.J.); heli.tiirola@uef.fi (H.T.)

**Keywords:** paediatric and young adult, patients, intervention, psychosocial, RCTs, logic model, review

## Abstract

**Background/Objectives**: Psychosocial interventions bring mental and social benefits to paediatric and young adult patients. Gaining insight into the optimal psychosocial intervention strategies and their process mechanisms can contribute to the deepening of practice in this field. **Methods**: This systematic review evaluates the psychosocial interventions that promote adaptability, resilience, and positive changes among paediatric and young adult patients. Following the Cochrane guidelines, the literature from 2000 to 2024 was reviewed, focusing on randomised controlled trials (RCTs). **Results**: Eighteen studies were included and analysed using a logic model framework. Therapeutic interventions that involved the reframing of cognition activities shaped personality changes, including resilience and adaptation, requiring significant investment, and they were influenced by individual characteristics and background. Process-oriented activities, such as art-, play-, and music-based therapies, improved emotional well-being and were affected by pain, cognitive abilities, and language skills. Behavioural changes are best achieved through interactive interventions, particularly group-based and parent-involved approaches, which improve social integration and physical abilities. **Conclusions**: Psychosocial interventions lead to positive changes in paediatric and young adult patients in terms of personality, emotion, and behaviour. Although the sample size for the behavioural changes is insufficient, understanding the mechanisms underlying these interventions benefits practice.

## 1. Introduction

Cancer is the leading cause of disease-related deaths among paediatric and young adult patients [1]. Paediatric and young adult patients are more susceptible to psychosocial, emotional, and mental problems compared to their healthy peers [2,3,4]. When these issues are not resolved during treatment, a significant number of survivors will experience depression, low self-esteem, anxiety, and attention problems compared to non-cancer controls [5,6].

Psychosocial interventions are therapeutic programs that incorporate techniques designed to improve health, functioning, and well-being by addressing psychological and social factors [7]. Therefore, they are key strategies for the alleviation of psychological symptoms, and they improve social functioning in paediatric and young adult cancer patients [8]. Additionally, they support the maintenance of holistic health, mitigating negative symptoms such as anxiety, depression, and stress disorder [9,10,11]. This approach promotes paediatric and young adult patients’ inherent personality strengths, such as resilience and adaptability [9,12,13,14]. Additionally, psychosocial interventions alleviate negative emotions (e.g., anxiety, depression, and stress disorder) [9,10,11] and enhance positive emotions [15,16,17]. Psychosocial interventions also help paediatric and young adult patients to improve their behaviour and academic performance [18]. Despite the numerous benefits of psychosocial interventions for paediatric and young adult patients undergoing cancer treatment, there is a need for studies with high levels of evidence to ascertain their impact on psychosocial health. Randomised controlled trials (RCTs) remain the gold standard for evidence in clinical research [19]. Therefore, it is essential to conduct a systematic analysis of the literature based on RCTs of psychosocial interventions.

In the current study, we combined systematic review and theory-driven (realist) logic model techniques to address the following three research questions: (a) Which psychosocial interventions and their targets have been utilised to enhance effective outcomes for paediatric and young adult cancer patients? (b) According to the logic model, what are the proposed mechanisms of inputs and outputs linked to these targets? (c) What are those psychosocial interventions’ main outcomes and contextual factors?

## 2. Materials and Methods

This systematic review has been registered with PROSPERO under registration number CRD420251022043, and is available at https://www.crd.york.ac.uk/PROSPERO/view/CRD420251022043 (accessed on 2 May 2025).

### 2.1. Search Strategy

A systematic review was conducted, according to the Preferred Reporting Items for Systematic Reviews and Meta-Analysis’s (PRISMA) 2020 (see https://www.prisma-statement.org/prisma-2020, accessed on 2 May 2025) checklist and flow diagram, from April 2024 to May 2024. Considering that the topic is an interdisciplinary field involving psychology, sociology, and medicine, as well as for the sake of professionalism and depth, the PubMed (including MEDLINE), PsycINFO, Web of Science, and Google Scholar databases were searched. In order to exclude outdated studies and consider the impact of technological changes on the field, the search was restricted to publications dated from January 2000 to January 2024. Using the Cochrane Population, Intervention, Comparison, and Outcomes (PICO) structure, Medical Subject Heading (MeSH) terms and keywords were used, including paediatric patients (“children” OR “adolescents” OR “teenager” OR “young adult”) with cancer (OR “oncology” OR “leukaemia” OR “lymphoma” OR “brain tumours”) AND intervention (OR “psychotherapy” OR “program” OR “strategy” OR “intervene” OR “clinical practice” OR “pilot” OR “therapy”) AND randomised clinical trials (OR “RCTs” OR “randomised trials” OR “randomised controlled studies” OR “controlled randomised trials” OR “randomised prospective studies”) AND psychosocial (OR “psychological” OR “social” OR “adjustment” OR “resilience” OR “quality of life” OR “emotion” OR “positive change”). Additionally, to ensure the quality and reliability of the data, non-peer-reviewed grey literature was not included. A snowballing method was used, and the references in all relevant studies and reviews were subjected to further examination.

**Figure 1:**
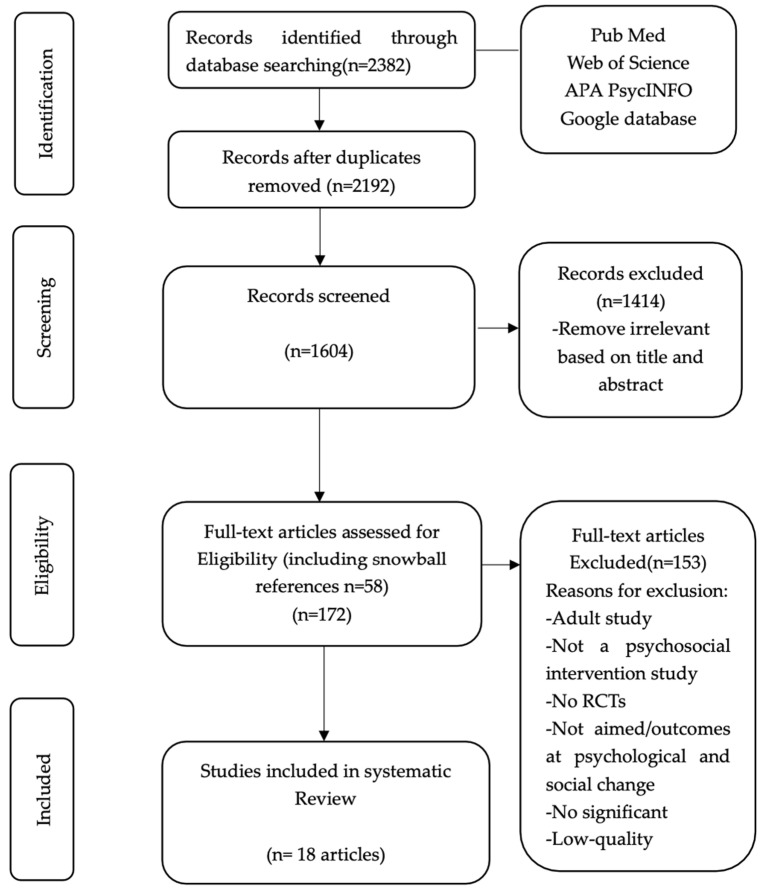
PRISMA flow diagram of study selection, inclusion, and exclusion.

The study selection process followed the predetermined inclusion and exclusion criteria. Initially, article titles identified through the search terms were screened to exclude irrelevant ones. Abstracts of the remaining articles were then reviewed, and those not meeting the criteria were excluded. Finally, the full texts of the remaining articles were analysed to make the final selection. Two researchers independently screened the literature according to the inclusion and exclusion criteria; then, they cross-checked their findings. If there were any objections, they consulted the evaluator and discussed any issues. Figure 1 shows the PRISMA flow diagram of study selection, inclusion, and exclusion.

### 2.2. Inclusion and Quality Assessment Process

The review was performed in accordance with the Cochrane guidelines [20]. The inclusion criteria for this study were as follows: (a) Population: This study included individuals under 25 years. Interventions could involve family members, parents, or siblings, but the focus was on a paediatric and young adult cancer patient. (b) Interventions: This review included psychosocial interventions to achieve psychological and social outcomes for paediatric and young adult cancer patients. (c) Outcomes: Beneficial psychosocial effects, such as adaptation, resilience, well-being, quality of life, personal development, hope, meaning, self-esteem, social support, optimism, gratitude, satisfaction, and family environment resulting from psychosocial interventions for paediatric and young adult cancer patients were described. (d) Study Designs: All responses included randomised controlled trials (RCTs), including randomised controlled studies, controlled randomised trials, and the follow-up study of RCTs. Two independent reviewers applied the predefined inclusion criteria to screen the records. In case of disagreements, a third reviewer was involved in the discussion to determine whether to include the record.

The exclusion criteria for this study were as follows: (a) case reports, review papers, book chapters, and conference abstracts; (b) studies where the age of participants was over 25 years; (c) trials that focused solely on outcomes for siblings and parents; (d) interventions aimed at reducing physical pain, symptoms and those involving pharmacological methods; (e) studies where no significant effect on psychosocial positive change was found; (f) and non-English publications. Two researchers followed the exclusion criteria and evaluated the full texts for inclusion eligibility. In cases of disagreement, a double-check process was used, and a quality assessment was conducted to determine whether the study should be included.

Quality assessment of studies. To evaluate the methodological quality and risk of bias of the randomised controlled trials (RCTs), the Cochrane Risk of Bias Tool 2.0 (RoB 2) [21] was used. This tool assesses five domains: (1) randomisation process (selection bias), (2) deviations from intended interventions (performance bias), (3) missing outcome data (attrition bias), (4) measurement of outcomes (performance and detection bias), and (5) selection of reported results (reporting bias). Each domain includes sub-domains with algorithmic results and assessor judgments to determine potential biases. Researcher X. L. conducted the assessment of the studies, while H. C. reviewed the evaluation. Studies with a risk of bias score higher than 8.5 (low risk) were included, and ultimately, 18 articles met the criteria and were included in this study.

### 2.3. Data Extraction and Analysis

Due to data limitations, a Logical Model Evaluation Framework (LMEF) was employed instead of a meta-analysis to provide a more tailored, context-sensitive, and comprehensive understanding of the mechanisms underlying psychosocial interventions. LMEF is widely used in evaluating health-related intervention programs [22,23,24,25,26,27]. It reflects on the strengths and weaknesses of their logical chains and aids in the future reference and improvement of interventions. A logic model visualises a system and is designed to highlight key components and their interrelationships within the system [28]. However, the logic model has not yet been utilised in the systematic review of psychosocial intervention evaluation for paediatric and young adult cancer patients. When evaluating psychosocial interventions, most views focus on effectiveness and outcomes [13,29,30] rather than on the process mechanisms underlying these intervention pathways. From a function perspective, it is crucial to understand how the research process impacts individual changes and predicts its role in the environment [31]. Since psychosocial intervention is the cumulative result of a complex research process [8], it is essential to visually map out intervention typologies and their expected intermediate and long-term psychosocial outcomes [28,32,33,34].

In addition to participants’ demographic characteristics (e.g., mean age, number of participants, current cancer patient or survivor status), the data extraction and analysis were conducted using a primary data extraction form created based on the 10-step process for logic mode [33]. This process included problem and background, defining targets, inputs (e.g., intervention type, funding/resource, therapy, provider, setting, control variable, agreement, measurement, and follow-up procedures), and outputs (covering sample size, activities, group or individual, parent participate, sessions, strategies), outcomes (short-term, mid-term, and long-term outcomes in personality, emotion, and behaviour), and contextual factors. The final logic model and synthesised results are presented in textual and graphical forms.

## 3. Results

### 3.1. General Features of Studies

All studies included cancer patients under 25 years. A total of 1232 participants took part in eighteen studies, with a mean age of 11.3 years. Fifteen studies included participants who were currently diagnosed with cancer [33]. Three studies reported interventions with cancer survivors [35,36,37], and they were follow-up reports from previous studies among the fifteen studies. The majority of studies were from the USA (10 studies, among them, one combined with Canada, and one combined with Canada and Colombia) [35,37,38,39,40,41,42,43,44,45], which was followed by China (3) [18,46,47] and Iran (2) [48,49]. There is one study each from Iraq [50], Thailand [51], and Israel [52]. All studies (*n* = 18) are randomised controlled trials (RCTs). Table 1 summarises the key characteristics of the studies included in this review, i.e., publication, date, country, intervention, participants, and follow-up.

Using the RoB 2 tool, all studies demonstrated a low overall risk of bias. In the randomisation process, only one study had a medium risk of simple randomisation [51], while the others used valid randomisation methods and blind procedures to control for bias. In “Deviations from intended interventions”, only one study employed a single-blind design, which might introduce performance bias [46]. Additionally, five studies showed missing and dropout data that could potentially affect the study results, but the missing data were mainly related to the condition of the participants, with a dropout rate below 20% and appropriate handling of the missing data, resulting in a low overall risk [35,36,38,39,51]. Furthermore, all studies used validated and reliable psychological measurement tools. Although there may be some subjectivity in the scales, the risk of bias remains low. Finally, none of the studies had selective reporting, and the analysis adhered to the predefined outcome measures with no unreported results or post hoc analyses, demonstrating low reporting bias risk. All studies had an average risk of bias score above 8.5 (out of 10), indicating a low risk of bias across the studies.

### 3.2. Psychosocial Interventions, Objects, Measurement, and Outcomes

Participants in the studies received various forms of interventions. In total, 15 interventions were implemented across 18 articles. All interventions are classified as follows: art and music therapy (*n* = 5), cognitive behavioural therapy (CBT) (*n* = 3), resilience-related therapy (*n* = 2), play-based therapy (*n* = 1), family-centred therapy (*n* = 2), massage and humour therapy (*n* = 1), and a wish-fulfilling intervention (*n* = 1). All studies had at least one control group, which included standard treatment as usual, a waitlist, and an alternative low-dose intervention, such as a loan and reading books instead of the intervention. Table 2 provides detailed information about psychosocial interventions that are effective for promoting psychosocial outcomes, including targets, therapy types, measurement methods, age groups, and main outcomes.

**Figure 2:**
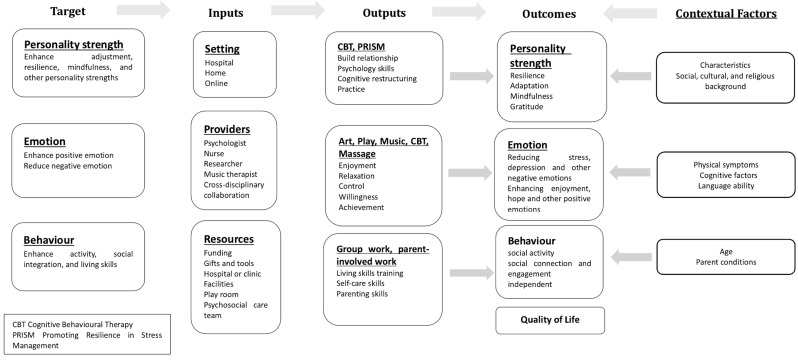
Proposed logic model illustrating putative links between intervention target, processes, and outcomes.

Following the logic model framework, we developed a logical analysis table for the interventions. This analysis helped us understand how the objectives are met and led to the creation of a logical framework diagram (see Figure 2). The text provides a detailed description of the inputs, outputs, outcomes, and contextual factors for each target.

### 3.3. Therapeutic Interventions Leading to Personality Changes

Out of six papers, four projects focused on cognitive issues and involved 381 cancer patients aged 8–25 (mean age = 15.2). The therapeutic activities included games, music creation, and educational training [35,36,39,40,46,53].

#### 3.3.1. Cognitive Reframing Influencing Personality Changes, Resilience and Adaption

The research findings indicated that cognitive reframing techniques used in cognitive behavioural therapy (CBT) and Promoting Resilience in Stress Management (PRISM) effectively improved psychological resilience [46,53] and adaptation [46]. These positive changes were attributed to activities that promoted positive cognition, such as creating smileys, using a happiness tree for feedback, and engaging in meaning-making activities [46,54]. In contrast, a virtual mind–body group program aimed at multiple resiliency factors showed significant improvements in gratitude and mindfulness but not in overall resilience outcomes [39]. This difference could be due to the emphasis on positive psychology skills rather than cognitive reframing. Additionally, the Therapeutic Music Video (TMV) intervention had limited short-term effects on personality changes, mainly impacting social behaviours like coping, social activity, and family dynamics [40]. This suggests that more comprehensive cognitive reframing activities may be needed for significant personality changes.

#### 3.3.2. Cognitive Reframing Also Brings Positive Changes in QoL and Emotion in the Long-Term

Cognitive reframing has been shown to bring about positive changes in quality of life (QoL) and emotions in the long term. Studies have demonstrated that cognitive reframing can significantly improve QoL [36,46,53] and reduce negative emotions such as stress, anxiety and depression. Additionally, cognitive reframing interventions have shown mid-term and long-term effectiveness in improving hope, distress and overall quality of life [36,54].

#### 3.3.3. Resource Consuming in Therapeutic Interventions

Therapeutic interventions require significant resources such as trained psychotherapists, coaches and psychologist. These interventions involve multiple sessions lasting 45 min to 2 h and require sustained engagement to build trust and enhance effectiveness. Follow-up periods of six months to five years have been supported by funding in some projects. Patients also need time post-intervention to accept their condition [53] and apply the skills learned [35,40].

### 3.4. Process-Oriented Activities Lead to Positive Emotional Changes

Process-oriented activities such as art therapy [50], play-based therapy [47,48], and music therapy have been effective in reducing emotional problems and improving quality of life. These activities focus on and the patient’s experience and the process rather than the end goal of change.

#### 3.4.1. The Sense of Control and Initiative Can Affect Emotions

Allowing patients to have control and make choices in their interventions has been shown to improve negative emotions and increase positive emotions [46,48,49,52]. Play and music activities have been effective in reducing negativity and enhancing positive emotions [40,41,50,53].

#### 3.4.2. Play and Music Activities: Reducing Negativity and Enhancing Positivity Emotions

Play-based occupational therapy and music interventions have been particularly effective in reducing anxiety and physical symptoms [46,49]. Listening to classical music enhances positive emotions, such as happiness and relaxation [38,51], whereas making music does not result in any significant changes in positive emotions [40].

### 3.5. Interactive Interventions Lead to Behaviour Changes

Interactive interventions have been successful in promoting behaviour changes in cancer patients. These interventions aim to improve patients’ behavioural abilities, adjust life plans, and enhance participation in daily activities [18,39,41,42,50].

#### 3.5.1. Group Work Enhances Patients’ Social Integration

Group work has been shown to enhance social integration and adaptation in patients. To ensure the effectiveness of the interventions, sub-groups of five or fewer participants were conducted to optimise patients’ engagement and help achieve integration goals [18,39,47]. Group work brings benefits, including social integration [18], social connection engagement [35,41,50], social support and coping [39], gain understanding [35], relationships with peers, and social activity [50].

#### 3.5.2. Parent-Involved Intervention Improves Patients’ Physical and Behavioural Abilities

Family-centred, parent delivery and parent participant are common ways of family-involved interventions for patients [18,41,42,50]. Parent-delivery active music intervention does provide patients with lower distress but increases the burden for parents in the short term [41]. However, research reveals patients’ physical and behavioural abilities, such as physical activity, health state, energy, sports, homework, and independent living, can be significantly enhanced by parent-involved interventions [18,50]. It is mainly due to these interventions that can enhance parents’ parenting abilities, thereby improving patients’ outcomes of physical and behavioural abilities.

### 3.6. Other Attributes to the Outcomes: Characteristics, Family and Social Environment

Characteristics and background influence patients’ personality change. Patients’ characteristics, such as cognitive abilities, language skills, personality, and psychosocial status [39,46,53], lead to different specific treatment outcomes depending on the individual. Secondly, the social, cultural, and religious background of the patients, particularly the illness perception of their culture influences their perspective [40,46,53].

Furthermore, physical symptoms, cognitive, and language ability have an impact on outcomes of emotional change. Physical pain and fatigue directly impact patients’ emotions, participation in interventions, and the effectiveness of emotional interventions [37,43,49,52]. Secondly, cognitive factors such as cognitive distortions, motivation, and resilience affect patients’ emotions [38,49]. For example, children’s reports may be inaccurate due to cognitive limitations [38]. Additionally, patients’ language ability and preference influence their willingness to participate, impacting the outcomes [38,41,47,50].

Finally, patients’ age and developmental status significantly affect their behaviour capabilities [18]. The intervention design should be age-appropriate for patients, as their motivation is influenced by the engagement level of activities, which in turn affects their acceptance [48]. In addition, parental conditions, hospital environments, and the stage of cancer treatment also impact the outcomes of interventions [18].

## 4. Discussion

### 4.1. The Value of Psychosocial Interventions for Patients

Even though psychosocial interventions primarily provide social and emotional benefits to children and young adults, their value extends beyond these areas. For example, resilience is enhanced through psychosocial interventions [46,53,54]. This is primarily due to the development of resilience therapy and increased resources [55,56]. The long-term benefits that psychosocial interventions can bring to children and young adults, including positive attitudes, finding meaning, spirituality, and self-transcendence, have been described [35].

Psychosocial interventions help alleviate children and young adults’ physical discomfort, improving their cooperation with medical treatment. Psychosocial interventions can not only positively affect children and young adults quality of life but also improve children’s energy/vitality, physical activity and overall health state [37,48,50,53]. Psychosocial interventions have also been shown to reduce negative features of the cancer journey, such as pain, fatigue, sleep disturbances, and disease symptoms [47,48,49].

### 4.2. Progressive Psychosocial Interventions’ Development

Based on the reviewed articles, there is a trend toward a more focused evaluation of the theoretical basis of psychosocial interventions for patients. Simultaneously, innovations have emerged in their implementation. Before 2017, theoretical applications were more diverse, including massage and humour therapy, family-centred interventions, listening to music, making a wish, and social adaptation [18,37,38,42,52]. However, since 2018, most interventions have focused on art and music therapy [35,50,51], play therapy [5,48], and cognitive behavioural and resilience therapy [36,39,46,49,53].

In addition, the style of these interventions has become more varied. Influenced by the digital age, more studies are exploring online programs and utilising internet platforms for communication and support activities [18,39,46,49,53]. Online positive psychological interventions can bring long-lasting and sustained changes to individuals’ psychological and social well-being, such as gratitude and mindfulness [39]. For example, computer-based CBT has developed from the original CBT model, enhancing its suitability and convenience for children and young adults [49]. There is also a growing trend in incorporating play or game elements into interventions, even if the primary focus is not on play therapy [35,41,47,50]. Notably, while CBT is limited in reducing anxiety, depression, and pain [30], its development through the incorporation of play and resilience has been shown to have positive outcomes, such as increasing resilience and adjustment [44,46].

### 4.3. Logic Model Enhances the Review

Logic models are highly effective in capturing and demonstrating the multifaceted and multidimensional impacts of complex interventions, including mediating and moderating variables. This is essential for understanding how interventions operate in different contexts [28]. The logical model allows a more flexible analysis, enabling researchers to select various relevant inputs and outputs when conducting a comparative analysis of results, thus observing the impact on the outcome more accurately. For example, the logic model clarifies that interventions targeting personality and the inherent traits of each child require professionals with a background in psychology, while emotional and behavioural interventions need personnel trained specifically in those areas. Additionally, projects with diverse objectives and a wide age range may find it more challenging to achieve their established goals. Therefore, we suggest that the design of psychological intervention content should consider the primary target and a limited age range. Finally, output is not necessarily proportional to investment but is closely related to the specific activity input. Therefore, some projects with low investment but high effectiveness are worth paying attention to and promoting, such as the Make-A-Wish program [52].

### 4.4. Limitations of Interventions and This Review

There are limitations to intervention studies, including a lack of long-term evidence for psychosocial outcomes in patients compared to their siblings and parents [45,57,58,59]. While play therapy, music therapy, and Make-A-Wish interventions show positive emotional impacts, long-term outcomes remain unclear due to limited data and funding for extended projects [35,40]. Psychosocial interventions require time to be effective, emphasizing the need for long-term investment [60]. Conducting longitudinal comparisons is challenging due to small sample sizes and significant differences in goals and measurements [37,43]. More long-term behavioural and emotional regulation interventions are needed to understand psychosocial impacts. Some intervention studies lack logical consistency, highlighting the importance of using a logic framework for future research.

This systematic review has limitations, such as the impracticality of conducting a meta-analysis due to the diverse intervention methods and outcome variables. Non-controlled trials were excluded, potentially missing valuable contributions. Non-English literature was not included, affecting the global representativeness. Grey literature was also not considered, leading to less comprehensive data support. Some studies had small sample sizes and experimental limitations, resulting in inconsistent results.

## 5. Conclusions

Psychosocial interventions positively impact patients’ personality strengths, emotions, and behaviour. This study shows that cognitive reframing activities in therapy lead to personality changes influenced by individual characteristics and background, requiring significant investment in high-quality providers and resources. Process-oriented activities, like art, play-based, and music therapies, significantly improve emotional well-being, offering diverse activities for children and young adults. Interactive interventions, especially group-based and parent-involved approaches, are effective in achieving behavioural changes. The logic model helps analyse effectiveness by aligning goals with professionals and emphasising cost-effective programs like Make-A-Wish. However, the multiplicity of study designs and lack of logical chains in some studies underscore the need for logical guidance in psychosocial intervention projects. There is a lack of randomised controlled trials (RCTs) to evaluate long-term outcomes, particularly in emotion and behavioural change domains, indicating the necessity for comprehensive long-term studies.

## Figures and Tables

**Table 1:** Summary of key characteristics of review studies.

Publication	Country	Design	Intervention	Period Sessions	Participants	Sample Size	Age Mean Age (MA)	Follow-Up	Overall Bias
Abdulah et al., 2018 [50]	Iraq	RCT	Painting and handcrafting-based group art therapy	Period: 1 month Sessions: 20	Patients	66	7–13 MA = 9.5	1 week	90
Hamedi et al., 2020 [49]	Iran	RCT	Conventional CBT and computer CBT	Period: 45-min Sessions: 6	Patients	45	9–12 MA = 10.5	Not specified	92
Kemper et al., 2008 [38]	USA	RCT	Listen to music	Period: 40-min Sessions: 2	Patients	63	0.8–17.9 MA = 9	Not specified	88 (missing data minimised)
Lester et al., 2020 [39]	USA, Canada, and Colombia	RCT	Virtual mind–body group program (RY-NF)	Period: 45-min Sessions: 8	Patients	51	12–17 MA = 14.37	6 months	92
Li et al., 2023 [47]	China	RCT	Child Life intervention	Period: 2 months, 30–40 min Sessions: 16	Patients	96	8–14 MA = 11	3 days	92
Lyon et al., 2014 [39]	USA	RCT	Family-Centred Advance Care	Period: 60-min Sessions: 3 weeks	Patients and parents	30	14–20 MA = 16.3	3 months	92
Mohammadi et al., 2021 [48]	Iran	RCT	Play-based occupational therapy	Period: 45-min Sessions: 8	Patients	25	7–12 MA = 9.28	Not specified	94
Phipps et al., 2010 [43]	USA and Canada	RCT	Therapeutic massage and humour/relaxation therapy	Period: 30-min 3 times per week Sessions: 4 weeks	Patients and parents	171	6–18 MA = 12.8	6 months	90
Phipps et al., 2012 [37] (follow-up of Phipps et al., 2010 [43])	USA and Canada	RCT	Therapeutic massage and humour/relaxation therapy	Period: 30-min 3 times per week Sessions: 4 weeks	Patients and survivors	97	6–18 MA = 12.8	24 months	88 (missing data handled)
Piasai et al., 2018 [51]	Thailand	RCT	Computer drawing–storytelling and guided imagination with classical music (GIM)	Period: 30-min drawing–storytelling and 30-min GIM	Patients	40	6–12 MA = 9.28	1 h	88 (simple randomisation)
Robb et al., 2014 [40]	USA	RCT	Therapeutic Music Video (TMV) intervention	Period: 3 weeks Sessions: 6	Patients	113	11–24 MA = 17.1	100 days	94
Haase et al., 2020 [35] (follow-up of Robb et al., 2014 [40])	USA	RCT follow-up	Therapeutic Music Video (TMV) intervention	Period: 3 weeks Sessions: 6	Patients and survivors	14	13–22 MA = 17	5 years	90
Robb et al., 2016 [41]	USA	Pilot RCT	Active Music Engagement (AME)	Period: 45-min Sessions: 3	Patients	16	3–8 MA = 5.5	30 days	92
Rosenberg et al., 2019 [53]	USA	RCT	Promoting Resilience in Stress Management (PRISM)	Period: 30-50 min within 2 months Sessions: 7	Patients	92	12–25 MA = 16.4	6 months	94
Rosenberg et al., 2021 [36] (follow-up of Rosenberg et al., 2019 [53])	USA	RCT	Promoting Resilience in Stress Management (PRISM)	Period: 30-50 min within 2 months Sessions: 7	Patients and survivors	57	13–25 MA = 16.4	24 months	92
Shoshani et al., 2016 [52]	Israel	RCT	Make a Wish intervention	Period: 5-6 months Session: 1	Patients	66	5–12 MA = 11	7 months	92
Yu et al., 2014 [18]	China	RCT	Family-centred Social Adaptation Capability (SAC)	Period: 20-60 min within 12 weeks Session: 4	Patients	240	3–7 MA = 5	Not specified	94
Zhang et al., 2019 [46]	China	RCT	Cognitive behavioural therapy (CBT)	Period: 5 weeks Sessions: 5	Patients	104	8–18 MA = 12.4	5 weeks	86 (lack of blinding)

**Table 2:** Psychosocial interventions that are effective for improving psychosocial outcomes.

Objects	Therapy	Intervention	Measurement	Age Group	Main Psychosocial Outcomes
Personality	Art and Music	Music video making (TMV)	Patient interviews	13–22	Reflection, self-expression, and finding meaning; overcoming distress; connecting with others; identifying personal strengths
			SDS, UIS, JCS-R, SPS, FAS, P-ACS, FSS, HRIS, RSTS ^1^	11–24	Courageous coping, social integration, and family environment
	CBT ^2^	CBT	CD-RISC, DASS ^3^	8–18	Improve resilience and reduce depression, anxiety, and stress
	Resilience-Based	Promoting Resilience in Stress Management	CD-RISC, PedsQL, GSCM, KPDS, HADS ^4^	12–25	Improve resilience, hope, cancer-specific quality of life, and reduce distress
		Virtual mind–body group program (RY-NF)	CAMM, MOCS-A, GQ-6, LOT-R ^5^	12–17	Gratitude, mindfulness, coping abilities and social support
Emotion	Play-Based	Child Life intervention	Information System, SDSC ^6^	8–14	Pain, anxiety, fatigue, and sleep disturbance
	Art and Music	Painting and handcrafting	Parent Interviews, KIHQ ^7^	7–13	Physical activity, mood, stress levels, and social engagement
	Art and Music	Drawing–storytelling and guided imagination	THFS, RS ^8^, Cortisol levels	6–12	Happiness and relaxation
	Art and Music	Active Music Engagement (AME)	Parent Interviews FA, PMSSF, IES-R ^9^	3–8	Emotional distress
		Listen to music	HRV, VAS ^10^	0.8–17.9	Relaxation
	CBT	CBT and computerised version	WBFPRS, CDI, STAEI, STAI ^11^	9–12	Reduced pain intensity, depression, anger, and anxiety
	Massage and Humour	Complementary interventions	CDI, PTSDI, CHQ, BFSC ^12^	6–18	Depression, PTSD, and quality of life, resilience
	Others	Make a Wish	BSI, PedsQLTM4.0, PANAS-C, HHI ^13^	5–12	Reduce distress, depression, and anxiety. Improve quality of life, hope and positivity
Behaviour	Play-Based	Play-based occupational therapy	TRSC, CPAS, FPS, VAS-F, FAS ^14^	7–12	Symptoms, pain, anxiety, and fatigue
	Family-Centred	Family-Centred Advance Care Planning (FACE-TC)	Patient report and interview SQ ^15^, Five Wishes	14–20	Reduce pain, anxiety, fatigue and sleep disturbance
	Family-Centred	Family-Centred Social Adaptation Capability (SAC)	Infants-Junior Middle School Student’s SAC Scale	3–7	Social adaption capability

^1^ McCorkle Symptom Distress Scale, Mishel Uncertainty in Illness Scale, Jalowiec Coping Scale—Revised, Reed Spiritual Perspective Scale, Family Adaptability/Cohesion Scale, Parent–Adolescent Communication Scale, Family Strengths Scale, Haase Resilience in Illness Scale, Reed Self-Transcendence Scale; ^2^ Cognitive–Behavioural Therapy; ^3^ Connor–Davidson Resilience Scale, Depression Anxiety Stress Scale; ^4^ Connor–Davidson Resilience Scale, Paediatric Quality of Life, Generic Short-Form and Cancer Modules, Kessler-6 Psychological Distress Scale, The Hospital Anxiety and Depression Scale; ^5^ The Child and Adolescent Mindfulness Measure, The Measure of Current Status—A, The Gratitude Questionnaire, The Life Orientation Test—Revised; ^6^ Sleep Disturbance Scale for Children; ^7^ The ‘KIDSCREEN-10 Index Health Questionnaire for Children and Young People’ (parent version); ^8^ The Happiness Face Scale, Relaxation Scale; ^9^ Facial Affect, Profile of Mood States—Short Form, Impact of Events Scale—Revised; ^10^ Heart Rate Variability, Visual Analog Scales; ^11^ Wong–Baker Faces Pain Rating Scale, Children’s Depression Inventory, State–Trait Anger Expression Inventory, State–Trait Anxiety Inventory; ^12^ The Children’s Depression Inventory, Posttraumatic Stress Disorder Reaction Index, The Children’s Health Questionnaire, The Benefit Finding Scale for Children; ^13^ The Brief Symptom Inventory 18, Paediatric Quality of Life Inventory, The Positive and Negative Affect Schedule for Children, Herth Hope Index; ^14^ Therapy-related symptoms checklist, Children Participation Assessment Scale, Wong–Baker Faces Pain Rating Scale, Visual Analog Scale—Fatigue, Faces Anxiety Scale; ^15^ The Satisfaction Questionnaire, Social Adaption Capability.

## Data Availability

No new data were created by this study.
